# “Skills for pills”: The dialectical‐behavioural therapy skills training reduces polypharmacy in borderline personality disorder

**DOI:** 10.1111/acps.13403

**Published:** 2022-02-04

**Authors:** Joaquim Soler, Elisabet Casellas‐Pujol, Isabel Fernández‐Felipe, Ana Martín‐Blanco, David Almenta, Juan C. Pascual

**Affiliations:** ^1^ Department of Psychiatry Hospital de la Santa Creu i Sant Pau Barcelona Spain; ^2^ Universitat Autònoma de Barcelona (UAB) Barcelona Spain; ^3^ Institut d’Investigació Biomèdica‐ Sant Pau (IIB‐SANT PAU) Centro de Investigación Biomédica en Red de Salud Mental, CIBERSAM Barcelona Spain; ^4^ 16748 Labpsitec Laboratorio de Psicología y Tecnología. Dpto. Psicología Básica Clínica y Psicobiología Universitat Jaume I Castelló Spain

**Keywords:** borderline personality disorder, deprescription, dialectical‐behavioural therapy, polypharmacy, psychopharmacology treatment

## Abstract

**Objective:**

Polypharmacy and overprescription of off‐label medications are common in patients with borderline personality disorder (BPD). The aim of the present naturalistic study was to explore whether the skills training module of dialectical‐behavioural therapy (DBT) can reduce polypharmacy in these patients in routine clinical practice.

**Methods:**

Retrospective, observational study of 377 patients with a primary diagnosis of BPD consecutively admitted to the BPD outpatient unit from 2010 through 2020. All patients were invited to participate in the DBT skills training module (DBT‐ST). DBT‐ST participants (*n* = 182) were compared with a control group who did not participate in DBT‐ST (*n* = 195). Pre‐post intervention changes in medication load and use of antidepressants, benzodiazepines, mood stabilizers, and antipsychotics were evaluated.

**Results:**

At baseline, most patients (84.4%) were taking at least one medication and 46.9% were on polypharmacy. Compared to controls, patients in the DBT‐ST group presented a significant reduction in the number of medications (2.67–1.95 vs. 2.16–2.19; *p* < 0.001), medication load (4.25–3.05 vs. 3.45–3.48; *p* < 0.001), use of benzodiazepines (54.4%–27.5% vs. 40%–40.5%; *p* < 0.001), mood stabilizers (43.4%–33% vs. 36.4%–39.5%; *p* < 0.001), and antipsychotics (36.3%–29.1% vs. 34.4%–36.9%; *p* < 0.001).

**Conclusions:**

These findings suggest that patients with BPD can benefit from the DBT‐ST module, which may reduce the medication load, particularly of sedatives. The results suggest that DBT‐ST may be useful to treat overmedication in patients with BPD and could help to promote “deprescription” in clinical practice.

**Significant outcomes**

- Most patients were on pharmacotherapy and polypharmacy was common.
- Patients who participated in the skills training module of dialectical‐behavioural therapy presented a significant reduction in the number of medications, medication load, and use of sedatives.
- Dialectical‐behavioural therapy skills training may be an effective strategy in helping to reduce polypharmacy and could promote deprescription in patients with BPD.

**Limitations**

- Because of the non‐randomized, retrospective study design, there is a risk of selection bias.
- The observed decrease in the medication load after skills training does not necessarily imply better clinical and/or functional outcomes.

## 1 INTRODUCTION

Borderline personality disorder (BPD) is a mental disorder characterized by a persistent pattern of emotional dysregulation, self‐image and interpersonal relationship instability, and marked impulsivity. It is a severe but common disorder, with an estimated prevalence ranging from 1.6% in the general population to 20% in psychiatric inpatient populations.^1^, ^2^ BPD is associated with severe psychosocial and occupational dysfunction, intensive use of mental health resources, and carries a substantial economic burden.^3^, ^4^

All of the main clinical guidelines support psychotherapy as the treatment of choice for BPD (for an overview see^5^, ^6^, ^7^). Although various psychological treatments have proven to be efficacious treatments for BPD, dialectical behavioural therapy (DBT) is the most frequently studied treatment, supported by robust empirical evidence from Cochrane reviews and meta‐analyses.^6^, ^7^ However, access to psychological therapies such as DBT is frequently limited, which is why most individuals with BPD only receive pharmacological treatment, even though no drugs have been specifically approved for the treatment of BPD and some clinical guidelines specifically recommend avoiding psychotropic drugs to treat BPD.^8^, ^9^, ^10^ While psychotropic drugs are commonly used to treat Axis I comorbidity disorders, they are also used to reduce the characteristic symptoms in patients with BPD such as intense mood instability, anger, and/or impulsive behaviour.^7^, ^11^, ^12^, ^13^ However, the optimal therapeutic drug for BPD‐specific symptoms is controversial, in part because of the scant research on pharmacological management in patients with BPD but also to the lack of consensus pharmacological recommendations in clinical guidelines for BPD.^5^, ^7^, ^12^

Several studies have shown that more than 80% of patients with BPD in western countries receive psychotropic drugs and more than 50% of patients receive polypharmacy.^11^, ^12^, ^13^, ^14^, ^15^ In a study conducted by our group, nearly three quarters of patients with BPD were taking antidepressants, 55% benzodiazepines, 40% mood stabilizers, and 35% antipsychotics.^15^ Some authors have described the risks of medication use in BPD, which include interactions with substances of abuse, addiction potential, increased risk of suicidal tendencies and disinhibition, serious side effects, a high risk for self‐injury with pills, and psychological risks such as diverting attention and energy from psychotherapeutic aims, thus altering the effectiveness of psychotherapy.^16^ The reasons for overprescription of psychotropic drugs in this population include: lack of perceived clinical alternatives; the prescription of drugs to address a temporary crisis that are never withdrawn (thus becoming chronic); attempts to treat all symptoms with medications; and treatment of comorbid disorders.^12^, ^16^, ^17^ Several strategies have been developed to reduce polypharmacy and promote quality pharmacological treatment, including efforts to familiarize physicians with the concept of "deprescribing" (defined as the process of reducing medications toward the minimum helpful treatment), to emphasize the need to prescribe drugs only on an "as needed" basis, and to establish a support group for physicians to avoid case overload.^16^

One approach that could be useful in deprescribing drugs in patients with BPD is psychotherapy. One of the most effective psychotherapeutic treatments for BPD is DBT to develop cognitive‐behavioural principles to replace maladaptive behaviours with healthier coping skills.^18^ DBT is an outpatient treatment supported by empirical evidence in BPD populations with other comorbid disorders such as post‐traumatic stress disorder, eating disorders, and substance abuse disorders (DBT‐S).^19^ Given that DBT is a multicomponent therapy, that is both expensive and time consuming, several studies have sought to identify the most relevant components of this therapy to reduce costs and treatment time. For example, the DBT skills training module (DBT‐ST) alone has proven to be effective in BPD.^20^, ^21^ DBT skills training comprises four modules focused on mindfulness, distress tolerance, interpersonal effectiveness, and emotion regulation.^18^, ^20^ Importantly, offering DBT to patients with BPD does not rule out the concurrent use of medications, which should be considered potential adjuncts to psychological therapy.^18^, ^19^ Nonetheless, DBT has been shown to promote the "skills for pills" effect, whose aim is to reduce the use of problematic drugs such as benzodiazepines, especially DBT‐S in patients with substance abuse disorders.^19^ The main idea is to taper the medication while simultaneously increasing the use and proficiency of the trained skills. In this way, physicians can encourage patients to learn and practice these skills, thus allowing them to reduce their use of prescription medications, when appropriate. Consequently, to ensure that pharmacological and psychological treatment are tightly coordinated, the clinician prescriber should ideally work closely with the psychology treatment team, which would also improve continuity of care.

### 1.1 Aims of the study

The aim of the present naturalistic study was to explore, in a naturalistic clinical setting, whether DBT‐ST reduces the use of medications and/or polypharmacy in patients with BPD. We hypothesized that providing patients with training in specific skills such as mindfulness, emotion regulation, and distress tolerance would reduce or eliminate the need for problematic drugs such as benzodiazepines.

## 2 MATERIAL AND METHODS

### 2.1 Participants

Data were retrospectively collected from 377 patients diagnosed with BPD and admitted to the outpatient BPD unit at the Department of Psychiatry at the Hospital de la Santa Creu i Sant Pau, between January 2010 and December 2020. This outpatient program is part of Spain's Public National Mental Health Service and provides specialized care for people with BPD referred from other psychiatric clinical units (eg, psychiatric emergency units, acute hospitalization units, general mental health outpatient services, private mental health centers, among others). A high percentage of these patients had previously received pharmacological and psychological treatment in other clinical units. Because of the lack of human and economic resources, general mental health public services in Spain have some limitations of offering a complete assistance to individuals with BPD. Compared with general mental health center, the BPD Unit offers: reliable confirmation of BPD diagnosis with validated instruments, greater accessibility to the unit, emergency attention in crisis, higher frequency and duration of visits, therapeutic team with specific experience and sensitivity for BPD, family care, psychoeducation of disorder, general management and non‐harmful strategies, and, finally, supervision of pharmacological treatment avoiding the excessive use of medication. Moreover, our BPD unit offers, as specific psychotherapeutic intervention for all individuals, groupal DBT skills training.

### 2.2 Study design

This was a naturalistic, retrospective cohort study. All participants underwent a clinical interview, and two semistructured diagnostic interviews were conducted by an experienced psychiatrist and a clinical psychologist. Admission to the BPD unit required a confirmed BPD diagnosis with the validated Spanish language versions of the Structured Clinical Interview for DSM‐IV Axis II Disorder (SCID‐II)^22^, ^23^ and the Diagnostic Interview Revised for Borderlines (DIB‐R).^24^, ^25^ Inclusion criteria were as follows: (a) age between 18 and 55 years; (b) primary diagnosis of BPD (DSM‐IV criteria) confirmed by structured interviews (SCID‐II and DIB‐R); (c) absence of comorbid psychotic or bipolar disorders; and (d) no neurological disease, intellectual disability, or any severe physical condition that could affect the psychotherapeutic intervention.

All study data were obtained from patient medical records, which included data relative to admission to the BPD unit (time 1) and after completion of the psychotherapeutic intervention and follow‐up in the BPD program (time 2). All patients included in this historical cohort in the BPD program were invited to participate in the DBT‐ST intervention. The cohort was divided into two groups: those who agreed to participate in the DBT‐ST intervention (DBT‐ST group; *n* = 182) and a control group consisting of those who did not participate in the intervention (control group; *n* = 195). Therefore, this was a non‐randomized, observational study.

In parallel with the psychotherapeutic intervention, all patients received periodic psychiatric evaluation and follow‐up visits, including supervision of pharmacological treatment to prevent excessive use of medications. Patients did not receive any other psychotherapeutic interventions. The psychiatrists who performed these periodic evaluations were not affiliated with DBT psychotherapy. The follow‐up period in the BPD program was variable and depended on the following factors: duration of therapy, whether or not the patient repeated the psychological intervention (DBT‐ST), and whether the patient dropped out during follow‐up.

The following sociodemographic and clinical data were collected: age; sex; follow‐up period (months); presence of lifetime comorbid axis I disorders (yes/no) classified into four groups: affective, anxiety, substance use, and eating disorders; DIB‐R total and subscale scores (affect [DIB‐aff]; cognition [DIB‐cog]; impulsive action patterns [DIB‐imp]; and interpersonal relationships [DIB‐per]).

Data on pharmacological treatment were obtained and classified as follows: antidepressants, including selective serotonin reuptake inhibitors (SSRIs), tricyclic antidepressants and dual‐acting agents; benzodiazepines; mood stabilizers; and first and second generation antipsychotics (FGA and SGA, respectively). For these drugs, we determined whether the participant was taking a given medication (yes/no) and the total number of medications (all categories) per patient.

The study adhered to the principles outlined in the Declaration of Helsinki and was approved by the Clinical Research Ethics Committee at the *Hospital de la Santa Creu i Sant Pau*. Written consent to participate in the study was not considered necessary as all data were collected retrospectively from routine admission data and subsequently anonymized. We checked the medical records of all patients to verify that no written objection to the use of this information was included in the record.

### 2.3 Instruments

The following instruments were administered to all participants.

#### 2.3.1 Structured Clinical Interview for DSM‐IV Axis II Disorder (SCID‐II)

The SCID‐II is a semistructured interview designed to assess DSM‐IV personality disorders. The Spanish validation study showed that this instrument discriminates well between personality disorders and has good inter‐rater reliability.^22^, ^23^

#### 2.3.2 Diagnostic interview revised for borderlines (DIB‐R)

The DIB‐R is an instrument designed to diagnose BPD and to assess the severity of the disorder within the last 2 years. The Spanish version has demonstrated good internal consistency (Cronbach's alpha, 0.89; sensitivity, 0.81; and specificity, 0.94).^24^, ^25^

#### 2.3.3 Medication load index

This index measures the total medication load. Following the method proposed by Hassel et al.,^26^ the total medication load index was calculated by coding the dose of each antidepressant, mood stabilizer, antipsychotic, and anxiolytic medication as follows: absent = 0, low = 1, or high = 2. For antidepressants and mood stabilizers, the doses and types were coded according to the method proposed by Sackeim.^27^ Antipsychotic doses were converted into chlorpromazine dose equivalents and coded using the mean effective daily dose as reference.^28^ The lorazepam dose was also coded to the reference of the midpoint of the recommended daily dose range from the Physician's Desk Reference.^29^ Scores for each of the individual medication were summed to create a total medication load index. The composite total medication load index seeks to reflect the dose and variety of the different medications taken. The index score is the sum of all individual medication codes in each medication category for each participant. We also calculated the sedative load index, which includes all medications that contribute to sedative load: benzodiazepines, antipsychotics, and drugs with a sedative component or side effect, including pregabalin, gabapentin, lamotrigine, oxcarbazepine, carbamazepine, topiramate, and mirtazapine. Only those medications that were regularly used by the patients were considered when comparing the indices at each time point.

### 2.4 Psychotherapeutic intervention

#### 2.4.1 Dialectical‐behavioural therapy skills training

The DBT skills training intervention is an adaptation of the DBT format drawn from one of the four intervention modes of the standard version.^18^, ^20^, ^21^ DBT‐ST consisted of weekly skills training sessions (120 min each) held over a 6‐month period. Upon completion of this 6‐month program, patients were invited to repeat the program to further reinforce the skills learned. All training sessions were conducted by two experienced psychotherapists, each with more than 10 years of clinical experience and specific training in DBT (Behavioral Tech Inc.). The treatment groups consisted of 9–12 participants. None of the participants received any other type of individual or group psychotherapy during the study period.

Dialectical‐behavioural therapy skills training aims to promote behavioural change, to help participants learn how to be interpersonally effective, and to learn how to better regulate emotions, and foster acceptance, mindfulness, and distress tolerance. The group sessions were all structured in a similar manner: teaching the content, in‐session practice of the strategies, and weekly homework assignments. The skills taught in a given week were reviewed at the following session.

The DBT skills training consists of four modules, as follows:

##### Mindfulness

This training module aims to help participants to develop non‐judgmental awareness, attentional control, and sense of self. Other aspects taught in this module include observing and describing thoughts, emotions, bodily sensations, and events; and fully engaging in experiences without judgment, focusing on one thing at a time, and being effective.

##### Distress tolerance

This module involves training participants to perceive the environment without demanding that it be different. Participants learn to experience and accept painful emotions without trying to change them. This module focuses on teaching strategies to help manage crises and skills to accept reality.

##### Emotion regulation

In this module, participants learn skills to help identify, label, and describe emotions, and to experience emotions from a mindful attitude to decrease vulnerability to unpleasant emotions, increase pleasant emotions. Participants learn to act in the opposite way when emotions are not justified.

##### Interpersonal effectiveness

This module contains strategies for solving problems at the interpersonal level. The focus is on teaching participants how to reach personal objectives while maintaining relationships and self‐respect. Participants also learn how to ask for what they need and on knowing how to say no when appropriate.

##### Control group

This group included all patients that did not participate in the DBT‐ST intervention for any reason, which included any of the following: scheduling conflicts (*n* = 48 [24.6%], mainly for academic reasons); work‐related issues (*n* = 53, 27.2%); preference for other types of therapy (*n* = 13, 6.7%); preference for individual therapy (*n* = 66, 33.8%); preference for private psychotherapy (*n* = 10, 5.1%); and others (*n* = 5, 2.6%).

Although these individuals did not receive any specific psychotherapeutic intervention for BPD, compared with general mental health services, they valued the higher frequency of psychiatric visits, attention in crisis, family care, and greater experience and sensitivity in the management of BPD. This follow‐up visits also include supervision of pharmacological treatment avoiding, if possible, the excessive use of medications, as recommended by all clinical guidelines. They also received non‐harmful strategies based on the *Handbook of Good Psychiatric Management for Borderline Personality Disorder*.^30^

### 2.5 Statistical analysis

Data were analyzed with the IBM‐SPSS Statistics for Windows, v. 25.0. All data were screened for skewness and kurtosis to test assumptions of normality. All hypotheses were tested to a two‐sided significance level of 0.05.

First, patient demographic and clinical characteristics at baseline (time 1) were described using typical measures of frequency, central tendency, and dispersion. To compare these characteristics between groups (DBT‐ST vs. control group), chi‐square tests (or Fisher's exact test if expected frequencies were <5) were used for categorical variables, and *t*‐tests for independent samples were used for continuous variables.

Second, to determine the impact of the group allocation on the use of medication, the percentage of withdrawal of each class of drug (antidepressants, benzodiazepines, mood stabilizers, and antipsychotics) at time 2 was compared between both groups (DBT‐ST vs. control group) using chi‐square tests (or Fisher's exact tests if expected frequencies were <5).

Finally, to evaluate the impact of treatment on pre‐post differences in the mean number of drugs taken per patient, and in the medication and sedative load indices, we conducted multivariate repeated‐measures ANOVAs. Treatment effects were assessed by entering each variable as dependent variables; time (pre‐ and post‐treatment) was entered as a within‐subjects factor, and group condition (DBT‐ST and control group) was entered as a between‐subjects factor.

Post hoc analyses were carried out when significant interactions were found. Effect sizes are reported by partial eta squared, with values up to 0.01, 0.06, and 0.14 considered small, moderate, and large, respectively.^31^

## 3 RESULTS

### 3.1 Sample characteristics

The final sample consisted of 377 patients with a primary diagnosis of BPD. Table 1 summarizes the sociodemographic and clinical characteristics of the participants. The typical patient profile was a 30‐year‐old female with moderate clinical severity (based on DIB‐R total score). Most patients presented at least one lifetime comorbid Axis I disorder, most commonly substance use and eating disorders.

**TABLE 1:** Baseline demographic and clinical characteristics of the sample with differences between groups

Variables	Total sample (377)	DBT‐ST (182)	Control group (195)	*χ^2^*	*t*	*p*
Age, mean (SD)	30.51 (8.5)	30.92 (8.0)	30.14 (9.0)			n. s
Females, *n* (%)	336 (89.1%)	168 (92.3%)	168 (86.2%)			n. s
Married/stable couple, *n* (%)	140 (37.1%)	68 (37.4%)	72 (36.9%)			n. s
Employed, *n* (%)	138 (36.6%)	72 (39.6%)	66 (33.8%)			n. s
Comorbidities						
Axis I comorbidity	266 (70.6%)	133 (73.1%)	133 (68.2%)			n. s
Affective disorders	87 (23.1%)	47 (25.8%)	40 (20.5%)			
Anxiety disorders	54 (14.3%)	26 (14.3%)	28 (14.4%)			
Eating disorders	116 (30.8%)	61 (33.5%)	55 (28.2%)			
Substance use disorders	133 (35.3%)	62 (34.1%)	71 (36.4%)			
DIB‐R total score, mean (SD)	7.24 (1.2)	7.46 (1.2)	7.05 (1.2)		−3.27	0.001
Pharmacological treatment						
Medications, mean (SD)	2.41 (1.7)	2.67 (1.7)	2.16 (1.7)		−2.00	0.003
0	59 (15.6%)	18 (9.9%)	41 (21.0%)			
1	67 (17.8%)	35 (19.2%)	32 (16.4%)			
2	74 (19.6%)	32 (17.6%)	42 (21.5%)			
3	85 (22.5%)	45 (24.7%)	40 (20.5%)			
4	48 (12.7%)	25 (13.7%)	23 (11.8%)			
≥5	44 (11.7%)	27 (14.8%)	17 (8.6%)			
Polypharmacy	177 (46.9%)	97 (53.3%)	80 (41%)	5.69		0.017
Antidepressants	271 (71.9%)	142 (78.0%)	129 (66.2%)	6.56		0.014
Benzodiazepines	177 (46.9%)	99 (54.4%)	78 (40.0%)	7.83		0.005
Mood stabilizers	150 (39.8%)	79 (43.4%)	71 (36.4%)			n. s
Antipsychotics	133 (35.3%)	66 (36.3%)	67 (34.4%)			n. s
Medication load, mean (SD)	3.83 (2.9)	4.25 (2.8)	3.45 (2.8)		−2.75	0.006
Sedation load, mean (SD)	2.24 (2.1)	2.49 (2.1)	2.01 (2.0)		−2.28	0.023

Abbreviations: DIB‐R, Revised Diagnostic Interview for Borderlines; n. s., not significant; SD, standard deviation.

At admission to the BPD program, 84.4% of the patients were taking at least one medication. The mean number of medications was 2.4 (range, 0–8), and nearly half of the patients were receiving polypharmacy (≥3 medications). Nearly three quarters of the sample (71.9%) were on antidepressants (mostly SSRIs), 47% on benzodiazepines, 40% on mood stabilizers, and 35% on antipsychotics (mostly SGAs).

### 3.2 Clinical and pharmacological differences between the DBT‐ST group and controls non‐DBT‐ST group

There were no significant between‐group differences in terms of sociodemographic characteristics or the prevalence of Axis I comorbidities. However, participants in the DBT‐ST group had higher mean DIB‐R total scores (7.46 vs. 7.05), indicating greater clinical severity (Table 1). There were also small but statistically significant differences between the groups at the timeframe (time in months between time 1 and time 2): DBT‐ST 11.1 (SD: 5) vs. Control group 9.77 (SD: 4.7; *p* = 0.007). We re‐analyzed using this variable as a covariate, without finding any influence on the results.

Participants in the DBT‐ST group were taking, on average, more medications than controls, with a higher proportion receiving polypharmacy, as well as higher medication and sedation load indices (Table 1). In addition, in the DBT‐ST group, a higher percentage of patients were taking antidepressants and benzodiazepines.

### 3.3 Differences between groups in prescription changes pre‐post intervention

**FIGURE 1:**
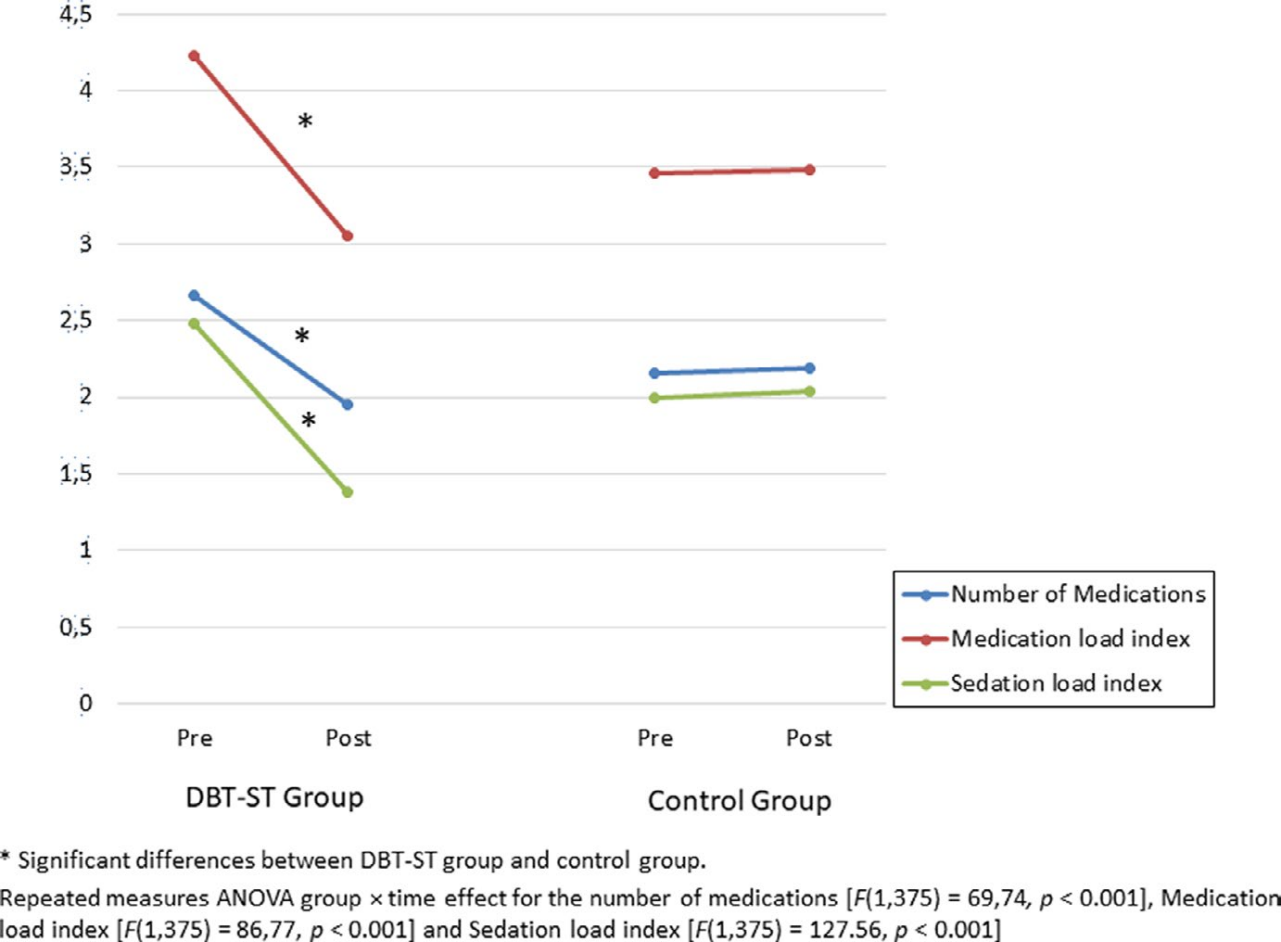
Differences between groups in prescription changes pre‐post intervention

**FIGURE 2:**
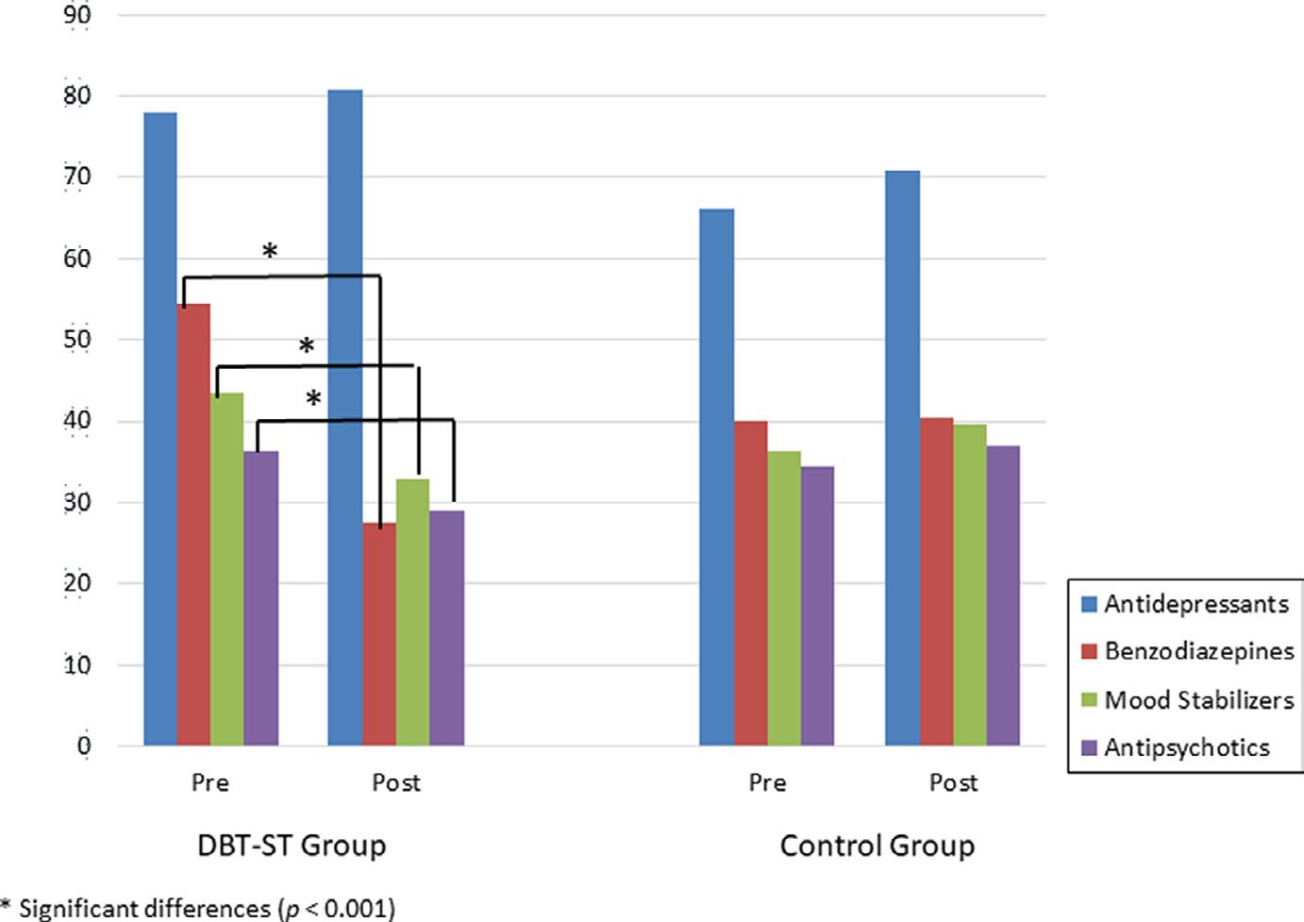
Pre‐post intervention differences in the prescription of antidepressants, benzodiazepines, mood stabilizers, and antipsychotics

Changes in prescription patterns are shown in Table 2 and Figures 1 and 2. Patients who participated in DBT‐ST intervention experienced a significant decrease in the number of medications prescribed [*F*(1,375) = 69.74, *p* < 0.001] and in the medication and sedation load indices [*F*(1,375) =86.77, *p* < 0.001; *F*(1,375) = 127.56, *p* < 0.001, respectively] over the course of the psychotherapeutic intervention compared with control group. The effect of intervention for all variables was large. Participants in the DBT‐ST group also significantly reduced their use of benzodiazepines (from 54.4% to 27.5%), mood stabilizers (from 43.4% to 33%), and antipsychotics (from 36.3% to 29.1%).

**TABLE 2:** Between‐group differences in changes in prescriptions pre‐post intervention

	DBT‐ST (182)		Control group (195)		*p* value
	Pre	Post	Pre	Post	
Number of medications, mean (SD)	2.66 (1.7)	1.95 (1.3)	2.16 (1.7)	2.19 (1.6)	<0.001
Medication Load, mean (SD)	4.23 (2.8)	3.05 (2.2)	3.46 (2.8)	3.48 (2.7)	<0.001
Sedation Load, mean (SD)	2.48 (2.1)	1.38 (1.6)	2.0 (2.0)	2.04 (2.0)	<0.001
Percentage of patients					
With medication	90.1	89.6	79	79.5	n. s
Antidepressants	78	80.8	66.2	70.8	n. s
Benzodiazepines	54.4	27.5	40.0	40.5	<0.001
Mood stabilizers	43.4	33.0	36.4	39.5	<0.001
Antipsychotics	36.3	29.1	34.4	36.9	<0.001

Abbreviations: DBT‐ST, dialectical‐behavioural therapy skills training; n.s., not significant; SD, standard deviation.

In the control group, no decrease in medication use was observed in any class of drugs. Moreover, for some medications, small increases were observed. The most notable finding was a significant decrease in the prescription of benzodiazepines, mood stabilizers, and antipsychotics in the DBT‐ST group versus controls.

## 4 DISCUSSION

The findings of the present study confirm previous reports that a high proportion of patients with BPD treated in clinical settings receive pharmacotherapy and polypharmacy. However, patients who received DBT skills training achieved a significant pre‐post reduction in the mean number of medications and in the medication and sedation load indices. These results confirm our main hypothesis that DBT skills training would help to reduce the medication burden. Compared to controls, patients who received DBT‐ST also significantly reduced their use of sedatives (benzodiazepines and antipsychotics).

In line with previous studies, we found that most patients with BPD were taking at least one medication, with nearly half of those patients receiving polypharmacy. This prescription practice in the public mental health system in Spain is consistent with clinical practice in other western countries^13^, ^14^ and higher than recommended in some clinical guidelines (eg, United Kingdom and Australian guidelines), which do not recommend prescribing pharmacological agents to treat BPD symptoms, but only to treat comorbid disorders or for short term use during periods of crisis.^7^, ^8^, ^9^, ^10^ Although clinical guidelines in other countries (Netherlands, Germany, and the United States) differ and have a more symptom‐oriented approach, all guidelines seem to agree that pharmacotherapy should not be used as a first‐line strategy because of the risks associated with these drugs.^32^

Interestingly, the patients who agreed to participate in the DBT‐ST intervention had higher DIB‐R total scores (indicating greater severity) and also took more prescription drugs than controls. Given the study design (observational, non‐randomized study), we hypothesize that patients with more severe BPD symptoms may be more motivated (ie, negatively reinforced) to change, and thus more likely to agree to participate in a skills training program to learn how to better manage their condition. This increased motivation to change also points to a greater interest in reducing pharmacological treatment. In this regard, findings from other studies have shown that individuals with greater levels of pretreatment distress were less likely to drop out of treatment, benefit more from DBT, and have better outcomes.^33^ In turn, this suggests that patients with less severe BPD may less motivated than those with greater severity to enroll in a weekly DBT program, which requires a strong commitment and high level of motivation.^34^

The findings of the current study suggest that DBT‐ST leads to deprescription in these patients, thus reducing the medication load, as we had hypothesized. The deprescription effect observed in patients who attended the DBT‐ST sessions is consistent with previous studies showing that DBT‐ST is an important component of the more comprehensive DBT program.^20^, ^21^ Those studies demonstrated the relevant role of this DBT modality in mediating reductions in suicide, depression, anger, and—at least partially—self‐harm, as well as in improving variables associated with psychopathology, mood, and affect. Those same studies have also shown that group DBT‐ST may even be more effective than individual DBT therapy alone.^20^, ^21^

Dialectical‐behavioural therapy is based on a biosocial model in which a skill deficit leads to the behavioural problems associated with BPD, such as parasuicide or aggressive behaviours, which are often a response to emotion dysregulation.^18^ Accordingly, one of the main objectives of DBT‐ST is to help patients to build a new behavioural repertoire, which should enhance emotion regulation and functioning. The strategies taught in the four DBT‐ST modules allow patients to better understand and regulate their emotions, better tolerate suffering, to more effectively respond on an interpersonal basis, and to participate mindfully and non‐judgmentally in life. The strategies taught in DBT‐ST allow patients to better understand their behaviours and thus to more effectively manage crises, by reducing emotional vulnerability and fostering the capacity to identify different emotional states to deploy respond more effectively to those emotional states. Skills training may empower the patients and increase self‐efficacy, thus consequently decreasing the number of medications, although this was not a goal *per se* of the intervention, but rather a consequence.

Although we observed an overall trend toward deprescription for some medications, this was not true for all prescription medications. For example, in both groups, antidepressant prescriptions were either unchanged or increased slightly, perhaps because of the prescribing clinicians' confidence in the effectiveness of antidepressants, leading them to exclude these from the deprescription goal. The preference to reduce sedative drugs may be explained because DBT skills training increases tolerance to distress, promotes coping and exposition, leading to habituation, extinction, or a change in the emotional experience, thus reducing dependence on medication as a main strategy for emotion regulation.

In some contexts, the use of certain medications (notably benzodiazepines and antipsychotics) allows the patient to avoid or escape from negative emotional states. Thus, when effective alternative strategies are available to achieve the same objective, this consequently leads to a reduction in medication intake. Not surprisingly, no changes in prescription patterns were observed in the control group, perhaps because these patients—who obviously did not receive DBT‐ST—may believe that symptom‐targeted pharmacological treatment is the only viable strategy to manage BPD and comorbid distress. Maybe, these patients were not looking for new strategies or skills and they preferred a complete assistance in the BPD unit with greater accessibility, attention in crisis, higher frequency and duration of visits, and family care. Indeed, it is also possible that non‐participation in DBT skills training influenced psychiatrists' practice because they were less motivated to reduce medication since patients did not have alternative tools to manage difficulties.

The most significant change observed in the study group was the marked reduction in the prescription of benzodiazepines, which decreased from 54.4% of patients at baseline to 27.5% post‐intervention. All major clinical guidelines advise against the use of benzodiazepines in BPD (except for short‐term use to address a specific, temporary crisis).^8^, ^9^, ^10^ Notwithstanding the clear recommendations against benzodiazepines, prescription rates for these pharmaceuticals remain high. Because of the addictive potential of these medications and their risk of increasing suicidal tendencies and disinhibition, an important goal in the psychiatric management of BPD is to reduce their use. These drugs are often prescribed in psychiatric emergency rooms to manage exacerbations or crises, as they can rapidly alleviate anger and/or negative emotions. In this regard, if patients received DBT‐based skills training to improve emotion regulation and distress tolerance, the use of these drugs would likely be reduced.

The prescription of mood stabilizers and antipsychotics, especially SGAs, for patients with BPD, has increased significantly in the last two decades,^15^ likely because of the promising results reported in clinical trials (especially for olanzapine, quetiapine, topiramate, and lamotrigine) in the treatment of affect dysregulation and impulse control.^7^ Antipsychotics have a sedative profile and could be an alternative to benzodiazepines. In any case, the application of DBT‐ST in patients with BPD would likely reduce the need for these drugs.

Dialectical‐behavioural therapy skills training could be a highly effective strategy to improve emotion regulation in patients with BPD. Unfortunately, DBT is not always available in the public health system, in part because it is a resource‐intensive treatment, requiring specific clinical training, staffing, and time. Consequently, it is essential to consider other efficient options that could reduce pharmacological strategies. In this regard, the application of the principles recommended in the Good Psychiatric Management model^30^ could help to reduce the medication load and provide alternative tools to help BPD patients cope with distress.

This study has several limitations. First, because of the non‐randomized, retrospective study design, there is a risk of selection bias. Although clinical guidelines recommend avoiding polypharmacy and unnecessary drugs in individuals with BPD, there may have been a more active deprescription activity in the DBT‐ST group compared to controls. It is also possible that there exist differences between groups regarding the motivation to reduce medication on both individuals and psychiatrists. Therefore, these findings will need to be confirmed in a prospective, double‐blind randomized clinical trial. Another potential limitation is that the observed decrease in the medication burden does not necessarily imply better functional outcomes. Consequently, other variables need to be included and evaluated in future studies to confirm that the reduced medication burden is because of clinical improvement and not because of other factors. Finally, uncontrolled clinical factors could also have influenced the results. By contrast, the main strength of this study is the naturalistic study design, which allowed us to analyze data obtained in the course of routine clinical practice, in contrast to other studies performed outside of real‐life clinical practice (eg, clinical trials). This design provides clinically relevant information since it was carried out in the context of usual psychiatric care in the Spanish public health system. Finally, the results of this study support the value of a coordinated and synergistic approach between psychopharmacologic and psychotherapeutic interventions in BPD.

In conclusion, the findings of this study suggest that DBT skills training appears to be an effective strategy to reduce polypharmacy in patients with BPD. Providing patients with tools to effectively manage distress could reduce the need for these patients to resort to the use of pharmaceuticals as their main approach to reducing distress. Further research into the role of deprescription in the context of skills training would be invaluable to better understand whether the decrease in medication load is followed by clinical and functional improvement.

## CONFLICT OF INTEREST

The authors declare that they have no conflicts of interest.

## AUTHOR CONTRIBUTIONS

JS and JCP conceived the study. AMB and JCP performed the statistical analyses. ECP and IFF drafted the first version of the manuscript. JS, ECP, and DA performed the psychotherapeutic intervention. All authors contributed to the writing and reviewing of the manuscript.

### PEER REVIEW

The peer review history for this article is available at https://publons.com/publon/10.1111/acps.13403.

## Data Availability

The data that support the present study are available from the corresponding author upon reasonable request.
